# Synthetic terpenoids in the world of fragrances: Iso E Super^®^ is the showcase

**DOI:** 10.3762/bjoc.15.252

**Published:** 2019-10-31

**Authors:** Alexey Stepanyuk, Andreas Kirschning

**Affiliations:** 1 Institute of Organic Chemistry and Center of Biomolecular Drug Research (BMWZ), Leibniz Universität Hannover, Schneiderberg 1b, 30167 Hannover, Germany

**Keywords:** asymmetric synthesis, fragrances, odorants, sandalwood, scents, terpenes, terpenoids

## Abstract

The history of fragrances is closely associated with the chemistry of terpenes and terpenoids. For thousands of years mankind mainly used plant extracts to collect ingredients for the creation of perfumes. Many of these extracts contain complex mixtures of terpenes, that show distinct olfactoric properties as pure compounds. When organic synthesis appeared on the scene, the portfolio of new scents increased either in order to substitute natural fragrances without change of olfactoric properties or to broaden the scope of scents. This short review describes the story of the most successful synthetic fragrance ever which is called Iso E Super^®^ as it is an ingredient in a large number of perfumes with varying percentages and is the first example being used as a pure fragrance. Structurally, it is related to natural terpenes like many other synthetic fragrances. And indeed, the story began with a classic in the field of fragrances, the natural product ionone.

## Review

“Iso E Super^®^ is to perfume what Tango Nuevo is to Tango Argentino” [1]

### Introduction – classical terpenes in perfumes

Perfumes (Latin “per fumus”, which means “through smoke”) have accompanied mankind for thousands of years dating back well before biblical times [2–3]. Plants and resins served as source for perfumes after alcoholic extraction. These extracts were not only used as fragrances but also as medicine (aqua mirabilis), aphrodisiac and elixir of life (aquavitae).

**Figure 1:**
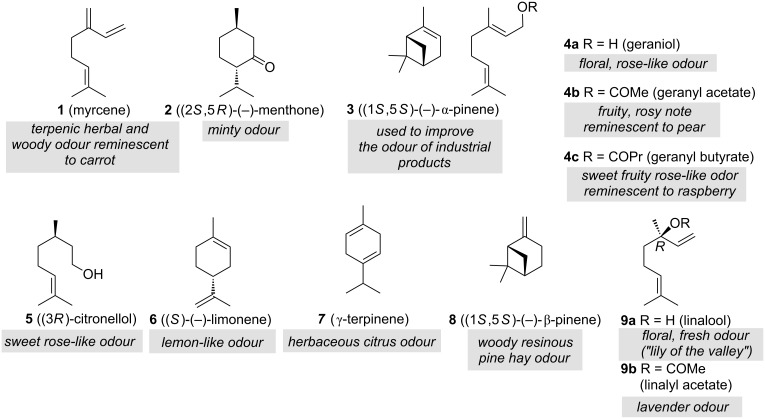
Terpene constituents **1**–**9** found in geranium and bergamot oils and specified odours of individual components. Perfumes are composed of compounds that are perceived immediately (top notes), that form the principal bouquet (heart notes) and compounds that are mainly perceived in the dry-out (bottom notes) [6]. Classification of 1. top notes: a) citrus, b) aldehydic, c) fruity, d) green and e) herbaceous/herbal, 2. heart notes: a) floral light, b) floral green, c) floral fresh, d), floral fruity, e) floral heavy and f) floral woody and 3. bottom notes: a) aromatic, b) balsamic, c) moss/leather/animalic, d) musk, e) amber and f) wood.

In 1882 *'Fougere Royale'* was created, a composition of coumarin, oak moss, geranium and bergamot, commercially launched by Houbigant [4]. The major constituents of geranium oil include myrcene (**1**), menthone (**2**), α-pinene (**3**), geraniol (**4a**), geranyl acetate (**4b**), geranyl butyrate (**4c**), citronellol (**5**), limonene (**6**) and linalool (**9a**). As for the bergamot oil monoterpenes limonene (**6**, 37%), γ-terpinene (**7**, 7%), β-pinene (**8**, 6%), linalool (**9a**, 9%) and linalyl acetate (**9b**, 30%) are key ingredients (Figure 1). The ratio of (*R*)-linalool and (*R*)-linalyl acetate (commonly >99.3% ee) is one of the quality indices as it affects the aroma of the essence of bergamot [5].

**Figure 2:**
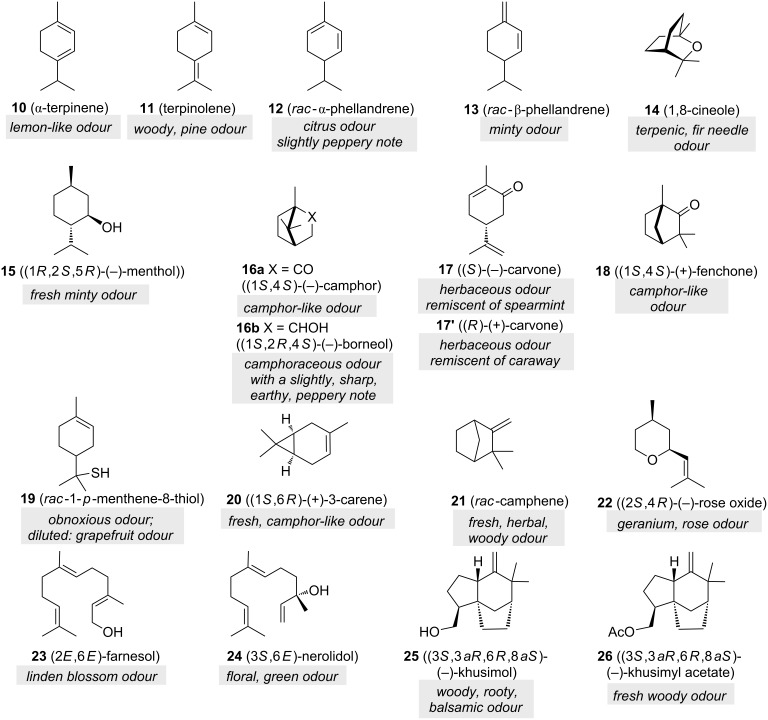
Other selected mono- and sesquiterpenes (**10**–**26**) as fragrance materials [6].

Nowadays, these historically important oils, rich in monoterpenes, are complemented by other essential oils from flowers, roots, fruit, wood, and moss [6], e.g., lavender and petitgrain oils are rich in linalyl acetate (**9b**) and lemon oil in γ-terpinene (**7**) and β-pinene (**8**). Commonly, essential oils are obtained by distillation with water or steam, and separation from the aqueous phase upon cooling. Many of these essential oils contain substantial fractions of mono- and sesquiterpenes, the most prominent examples and their olfactory properties being shown in Figure 2. α-Terpinene (**10**) is found in cardamom and marjoram oils, while isomeric terpinolene (**11**) is present in pine oils. α-Phellandrene (**12**) is a constituent of elemi oil, whereas red/pink pepper oils are rich both in α- phellandrene (**12**) as well as β-phellandrene (**13**). Rosemary and eucalyptus oils contain the monoterpene ether 1,8-cineole (**14**) and camphene (**21**) can be isolated from the Siberian fir needle oil. Cymbopogon oils provide among other components borneol (**16b**), geranyl acetate (**4b**) and citronellol (**5**). Besides limonene (**6**), (−)-carvone (**17**) is one of the main constituents in caraway oil and dill seed oil yields (+)-carvone (**17’**). Not surprisingly, camphor oil is rich in camphor (**16a**). Cypress oil yields 3-carene (**20**) as one major constituent. Mint oils serve as one possible source for menthone (**2**), menthol (**15**) and (−)-carvone (**17**). Essential oils collected from eucalyptus are rich in 1,8-cineole (**14**) and from fennel oil in fenchone (**18**). Farnesol (**23**) is present in many essential oils such as citronella, neroli, cyclamen and lemon grass. Nerolidol (**24**) is present in neroli, ginger, jasmine, lavender, tea tree and other essential oils. Finally, vetiver oil contains the sesquiterpene khusimol (**25**) from which the acetate (**26**) can be prepared by semisynthesis [6].

Obviously, nature served as starting point and guideline for creating scents and these lists reveal, that terpenes, particularly mono- and sesquiterpenes, have played a rather dominant role in the fragrance industries [6]. Over the last decades, the demand for fragrances has grown dramatically, so that plantations serve to provide the raw materials. In parallel, synthetic efforts also dramatically expanded to fulfil the huge demand of the consumer markets, new olfactory experiences included. Indeed, synthetic compounds were not introduced until the dawn of 19th century and first and foremost coumarin played a key role, first synthesised by Perkin in 1868 [7–8]. Following this breakthrough, many other perfumes were created based on synthetic molecules born from the newly established discipline synthetic organic chemistry. This made odorants available for the broad masses and perfumes to be worn according to one’s daily mood [2–3].

Key enabling milestones were the musk ketone accidentally discovered in 1894, being an important compound not derived directly from nature. Other musky compounds are (−)-(3*R*)-muscone, isolated in small-yields from glandular secretion of the musk deer, and 15-pentadecanolide were utilised too [9].

### The discovery and modern applications of Iso E Super^®^

**Figure 3:**
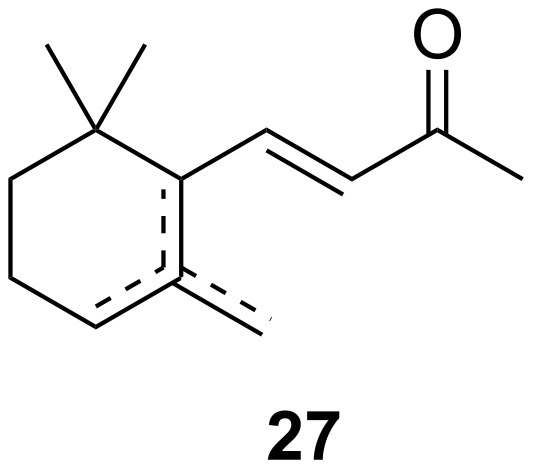
Main constituents of natural iris oil: irone (**27**).

It has to be stressed that musky odours were not the only scents of interest but also the spectrum of fragrances from violet flower oils. In fact, these were the most expensive of all available essential oils. Exorbitant quantities of flower petals were extracted to collect the oil, used directly in cosmetic formulations or spread on laundry to generate a characteristic smell. As for musk fragrances, there was a quest in the perfume industries to find a synthetic solution to create scents that mimic violet flower oils. First, a similarly smelling but more affordable orris root oil (*Iris pallida* Lam., fam. Iridaceae) was chosen for structural analysis. Thiemann and Krüger isolated irone (**27**, Figure 3), whose molecular formula was first falsely assigned as C_13_H_20_O [10].

**Scheme 1:**
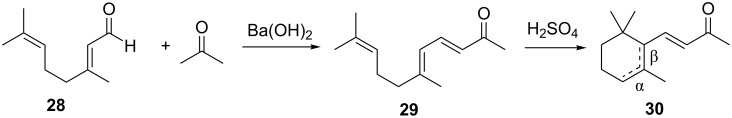
First synthesis of ionone (**30**) [11].

In an attempt to recreate this compound by condensation of acetone with citral (**28**) a compound with “a strange but not very characteristic odour” was formed, later named pseudoionone (**29**, Scheme 1). It turned out not to be suited for further investigations. However, after cleaning the glassware with sulfuric acid, a distinctive scent of violets was noted which later was linked to ionone (**30**) being created in the acidic medium. Thus, the category of synthetic ionone (**30**) and woody smelling compounds was born in 1893 and investigated further in the following years [10–13].

**Scheme 2:**
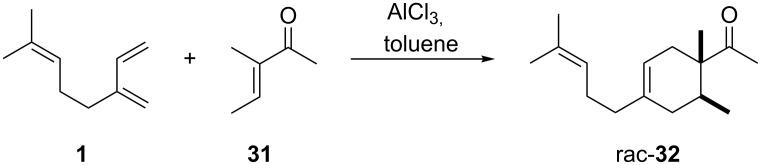
First synthesis of Ambrelux (**32**) [14].

**Scheme 3:**
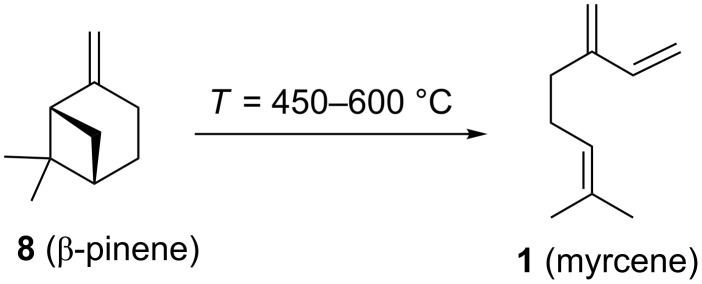
Industrial synthesis of myrcene (**1**) by pyrolysis of β-pinene (**8**).

Following this invention, many derivatives were produced to find new viable targets. These studies mostly focused on Diels–Alder cycloadditions to create structures that resemble terpenoids readily available from easily accessible and affordable starting materials like myrcene (**1**). One of the newly found products was Ambrelux (**32**, Scheme 2) that was further cyclised in a similar fashion previously mentioned for ionone compounds. This process yielded Isocyclemone E^®^ (**33**), later rebranded to the famous name Iso E Super^®^ (**33**) that is valid until today [9,14–15]. Indeed, myrcene (**1**) is one of the most versatile monoterpenes to be used as starting material for generating products in various industries. These include polymers, insect repellents, vitamins, flavours and fragrances [16]. Commercially, it is obtained from turpentine, a side product in paper manufacturing. Its main constituents are α-pinene (**3**) and β-pinene (**8**), 3-carene (**20**), limonene (**6**) and camphene (**21**). Since no large-scale source for myrcene (**1**) was available, a short route from readily available monoterpenes was established. Under pyrolytic conditions β-pinene (**8**) as constituent of turpentine undergoes a rearrangement to myrcene (**1**) (Scheme 3) [17–21].

**Scheme 4:**
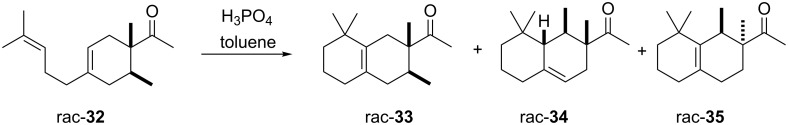
First synthesis of Iso E Super^®^ (**33**), Iso E Super Plus^®^ (**34**) and Georgywood^®^ (**35**) as a mixture of isomers [15].

To produce Ambrelux (**32**), myrcene (**1**) is reacted with dienophile (**31**) in a Diels–Alder cycloaddition promoted under Lewis-acidic conditions. In order to obtain Iso E Super^®^ (**33**), Brønstedt acid-mediated cyclisation, similar to the one utilised for the first synthesis of ionone (**30**), proved feasible on large scale. As it turned out, not only the one depicted, but several other cyclisation products formed. The main constituent was Iso E Super^®^ (**33**). A minor byproduct is now referred to as Iso E Super Plus^®^ (**34**, Scheme 4). Small modifications of the reaction conditions yielded other geometric isomers. In 2007, a thorough study was published by Fráter et al*.* disclosed of how such variations of parameters affect product formation and composition [22].

Interestingly, Iso E Super^®^ (**33**) itself shows a comparably high odour threshold of 500 ng L^−1^ as was reported in the original patent [15]. An impurity of ca. 5%, now called Iso E Super Plus^®^ (**34**), was made responsible for the characteristic smell having an odour threshold as low as 5 ng L^−1^ [23]. Naturally, this impurity was thoroughly analysed in the laboratories of Givaudan SA and finally secured in a patent as Iso E Super Plus^®^ (**34**). Later, also the second impurity Georgywood^®^ (**35**) with a higher odour threshold of 15 to 30 ng L^−1^ but better odour characteristics was patented [17–20]. Further details on the individual components of this complex mixture are listed in Table 1.

**Table 1:** Individual components of the complex Iso E Super^®^ mixture.

Component	Commercial name	CAS number	Odour (threshhold)

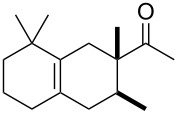 *rac*-**33**	Iso E Super^®^ (**33**)	59056-94-9	woody, floral, ambergris, violet, old-wood, lemony (500 ng L^−1^)
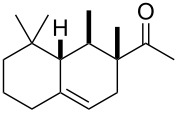 *rac*-**34**	Iso E Super Plus^®^ (**34**)	140194-26-9	woody–ambery, very strong odour (5 pg L^−1^)
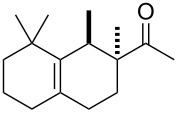 *rac*-**35**	Georgywood^®^ (**35**)	185429-83-8	(15 pg L^−1^–30 pg L^−1^)
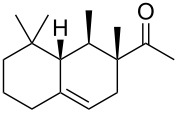 (+)-**34**	(+)-E Super Plus^®^ (**34**) (Corey also referred to it as “arborone”)	356088-93-2	intense woody odor, clean and pleasant (5 pg L^−1^)
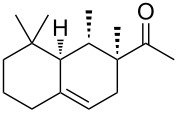 (−)-**34**	(*−*)-E Super Plus^®^ (**34**)	356088-90-9	faint odor
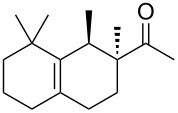 (−)-**35**	(*−*)-Georgywood^®^ (**35**)	828933-31-9	woody-ambery. bottom note: fresh, minty, green, sweet (20 pg L^−1^)
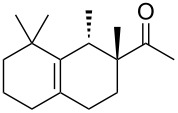 (+)-**35**	(+)-Georgywood^®^ (**35**)	828933-41-1	weakly woody bottom note: unpleasant, acrid, musty (3.5 ng L^−1^)
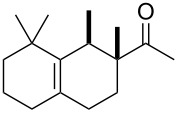 *rac*-**53**	**53**	260792-30-1	n.a.

It must be noted that the conditions for the synthesis of all Iso E Super^®^ related compounds vary slightly. The main difference lies in a prolonged isomerisation process of the Diels–Alder product **32** before and after the second cyclisation step. Georgywood^®^ (**35**) named after Georg Fráter is industrially produced with, e.g., methanol as additive to enforce isomerisation and suppress premature cyclisation [24–26].

Today, Iso E Super^®^ (**33**) and its isomers are widely used in a variety of perfumery products. From Haliston *Woman* that only contains a very small portion of this component and Christian Dior´s *Fahrenheit* consisting of 25% Iso E Super^®^ (**33**), LancÔme *Trésor* (18%) and Shiseido´s *Feminite du Bois* (43%), *Bois de Violette*, *Bois et Fruits*, *Bois et Musc*, *Un Bois Sepia*, *Un Bois Vanille*, Christian Dior´s *Dolce Vita*, just to mention a few (Table 2). Only recently, it was probed, whether it is possible to further increase the amount of Iso E Super^®^ (**33**) in a commercial perfume [25–26].

**Table 2:** Top fragrances with regard to their volume percentage (listed down to about 20%; the large number of perfumes with lower percentages are not listed) of Iso E Super^®^ in perfume oil [1].

Fragrance Name	Company	Launch year	Iso E Super^®^ (**33**) and Iso E Super Plus^®^ (**34**)

*Molecule 01*	Escentrıc Molecules	2005	100%
*Perles*	Lalique	2007	80%
*Orb_ital*	Nomenclature	2015	75%
*Poivre Samarcande*	Hermès	2004	71%
*Escentrıc 01*	Escentrıc Molecules	2005	65%
*Terre d’Hermès*	Hermès	2006	55%
*Incense Kyoto*	Comme des Garçons	2002	55%
*Incense Jaisalmer*	Comme des Garçons	2002	51%
*Fierce for Men*	Abercrombie & Fitch	2002	48%
*Kenzo Air*	Kenzo	2003	48%
*Encre noire*	Lalique	2006	45%
*Feminite du Bois*	Shiseido	1992	43%
*Fahrenheit*	Christian Dior	1988	25%
*Tresor*	Lancome	1990	18%
*Aventus*	Creed	2010	18%

This trend culminated in Schön´s creation of *Molecule 01* in the year 2005, a perfume that contains nothing else than Iso E Super^®^ (**33**). *Orb_ital* from Nomenclature (75% Iso E Super^®^) followed in the year 2015. This fragrance collection has set itself the task of using a range of synthetic fragrances as “overdoses” in perfumes.

**Figure 4:**
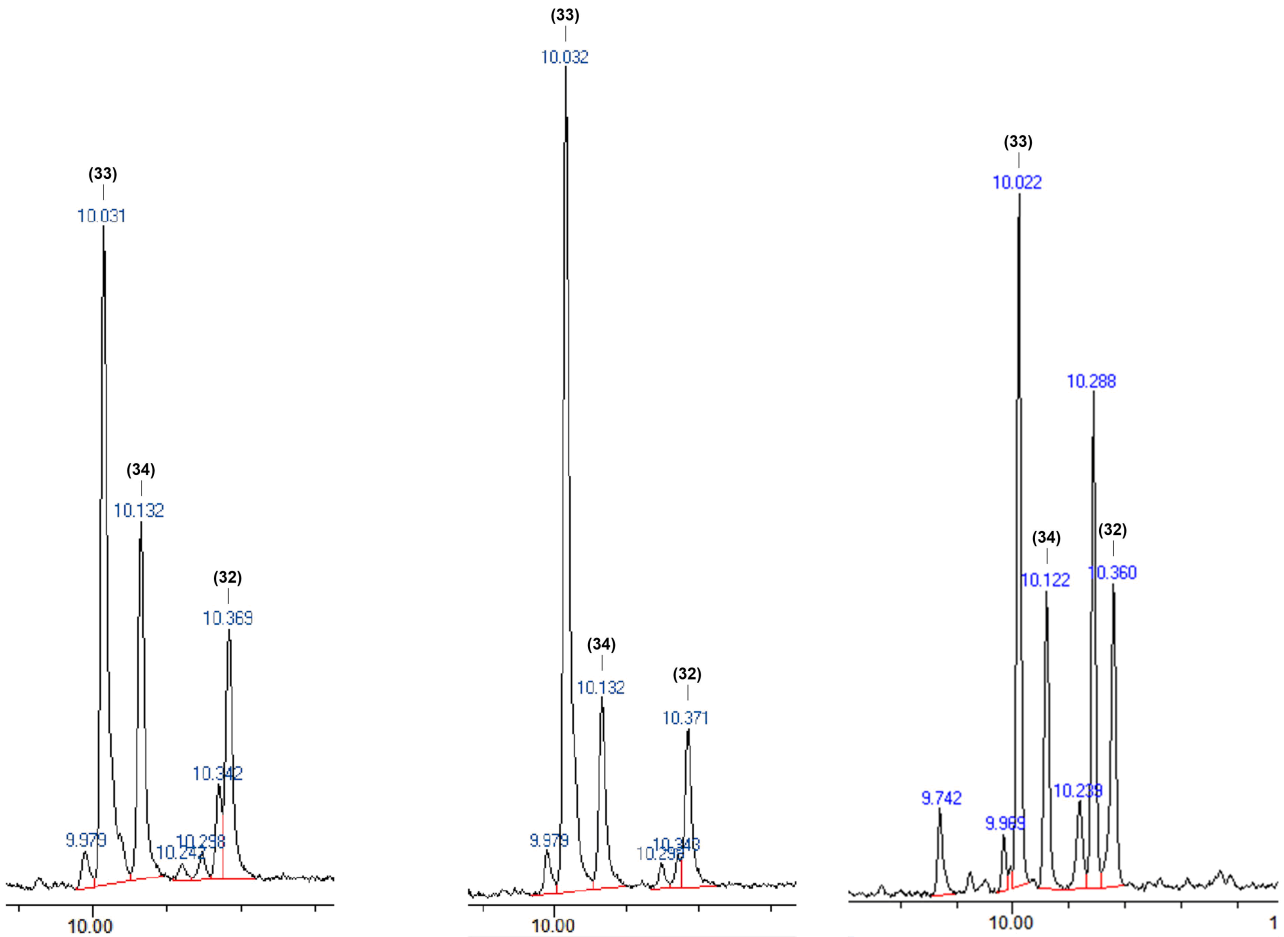
Iso E Super^®^ region of GC spectra of *Molecule 01* (left, 75 €–100 € per 100 mL; march 2019), a low-priced counterpart from TOP2BASE (middle, 20 € per 100 mL) and product produced in our laboratories under flow conditions. Note, although both samples provide a similar smell, the olfactory effects slightly vary because of different proportions of products and isomers produced in very small amounts that impact the nuances of a scent significantly (GC analyses were conducted in the author´s laboratories on a Zebron™ phenomenex ZB-1MS F&F (20 m, 0.18 mm, 0.18 µm) column).

The name *Orb_ital* derives from Orbitone, a brand name from the olfactory active (2*R*, 3*R*)-Iso E Super^®^ (**33**) [27]. It has to be stressed, that all compounds related to Iso E Super^®^ are not handled as single isomers but rather as varying mixtures because none of the industrial syntheses is very stereo- and regioselective as shown by GC analysis in Figure 4. So far efforts in industrial production have been directed towards product mixtures that are dominated by one isomer with favourable olfactory properties.

What seems to be counterintuitive for purely synthetically oriented or medicinal chemists, can be rationalised, when briefly considering the biochemical mechanism of the smell and the operation of scents. The odour impression is created by olfactory receptor neurons inside the nose. Since olfaction is a very complicated and broad field, it is hard to predict how molecules and mixtures of different molecules affect the perception. This is especially complex since odour impressions may change when concentrations are altered. On the lowest level, compounds of interest interact with so-called G-protein receptors consisting of seven intermembrane domains [28]. The quaternary structure including the membrane set up the active site. Approximately 370 different G-type proteins are known, that are linked with the odour perception. Because molecules can bind to an array of olfactory receptors generating a complex odour impression, an exact determination which proteins are linked to which smells or molecules is a very ambitious task. Hence, studies towards understanding interactions led to a Nobel Award in 2002 [29–31]. Even today correct modelling and protein crystallisation are immense challenges to be solved. Hydrophilic and hydrophobic interactions with the unpolar lipid layer make the tendency to yield suitable crystals even more difficult. Nevertheless, Palczewski and co-workers were able to crystallise the first GPCR (G-protein-coupled receptor) in 2000 confirming the previously described structure [28,32].

### Synthetic aspects of individual Iso E Super^®^ components

**Scheme 5:**
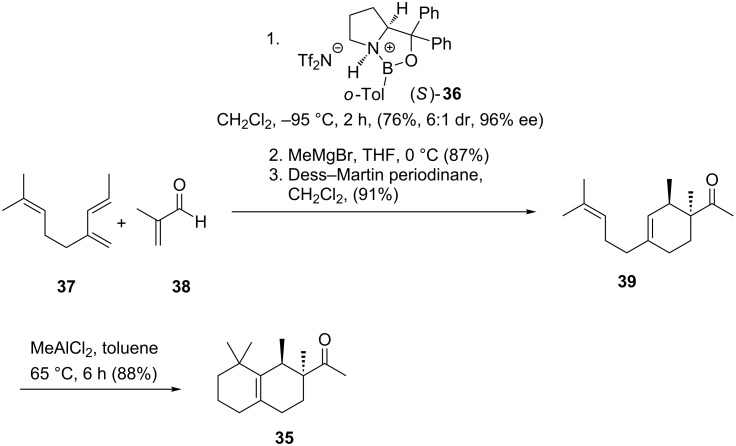
First synthetic route to (*−*)-Georgywood^®^ (**35**) by Corey and Hong [33].

The first target-specific synthesis of (−)-Georgywood^®^ (**35**) utilised the (*S*)-Corey–Bakshi–Shibata catalyst (**36**) for the enantioselective Diels–Alder cycloaddition (Scheme 5). The corresponding enantiomer (+)-Georgywood^®^ (**35**) was also prepared using the corresponding (*R*)-CBS catalyst (**36**).

**Scheme 6:**
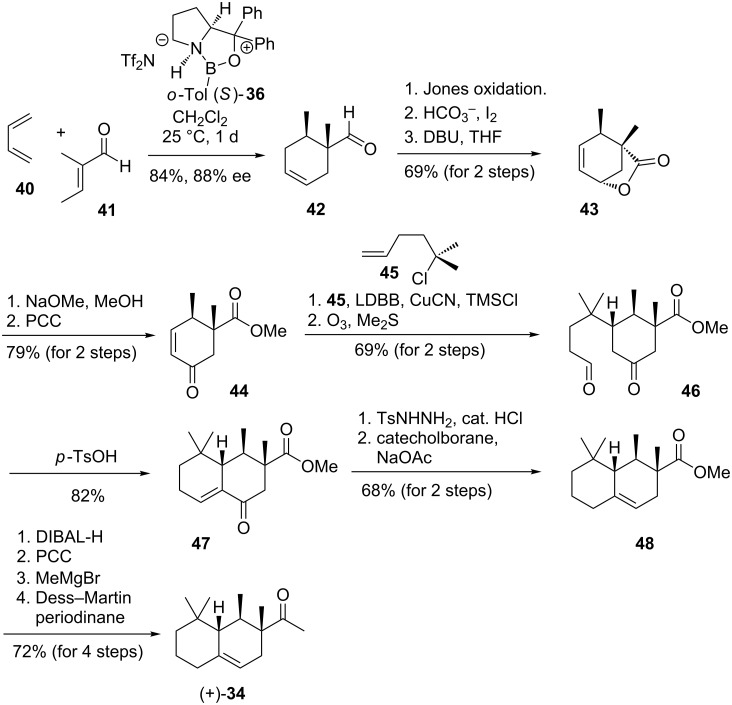
First synthetic route to the odour-active (+)-enantiomer of Iso E Super Plus^®^ (+)-**34** [33].

In contrast, the enantiomer (+)-Georgywood^®^ (**35**) was found to possess a relatively weak odour which was described as distinctly unpleasant and acrid-musty by several members of the Corey group [33]. The same approach led to the discovery of (+)-Iso E Super Plus^®^ (**34**) as a highly active component (Scheme 6). Fráter et al. confirmed these experiences after isolation of active olfactory compounds of Iso E Super Plus^®^ (**34**) and Georgywood^®^ (**35**). Racemic resolution provided a crystalline material that served to obtain an X-ray structure of the oxime derivative of (−)-(1*R*,2*S*)-Georgywood^®^ ((−)-**35**) [33–34].

Corey´s asymmetric synthesis of Iso E Super Plus^®^ ((+)-**34**) is initiated by a stereoselective Diels–Alder cycloaddition utilizing the CBS catalyst (**36**) to yield the cyclohexene derivative **42** with good facial selectivity [33]. Oxidation, iodocarboxylation and elimination yielded the lactone **43**. A series of functional group manipulations provided enone **44**, which underwent a cuprate-mediated Michael addition and liberation of the aldehyde **46** upon ozonolysis. After intramolecular aldol condensation the resulting enone **47** was transformed into cyclohexene **48** with shifted olefinic group by means of a reductive variant of the Wolff–Kishner deoxygenation. A straightforward four-step sequence finally yielded Iso E Super Plus^®^ ((+)-**34**).

**Scheme 7:**
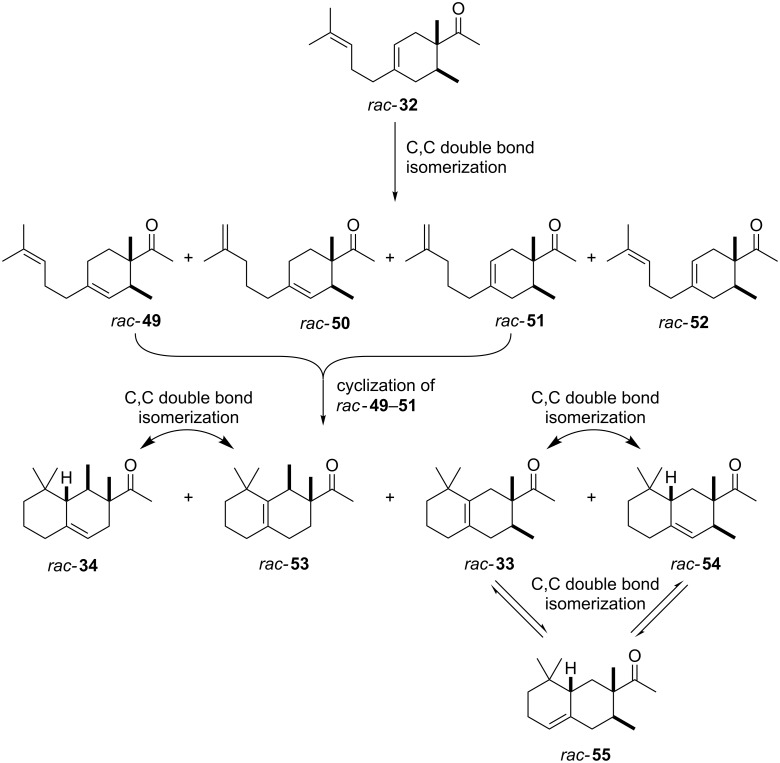
Analysis of the isomerisation process and formation of products. Most importantly, Iso E Super^®^ (**33**), Iso E Super Plus^®^ (**34**) and Iso Gamma (**55**) are formed [1]. All compounds are obtained as racemates [25].

Industrially pursued syntheses do not involve a specific stereoinducing step. In fact, it is mentioned in the patents that the standard industrial process of Iso E Super^®^ (**33**) utilises technical grade chemicals for both synthetic steps. The mixture of resulting isomers is then used in perfumes, when the smell meets standard criteria by quality control [14]. As encountered earlier, the second step of production is the most important one for product formation and composition. Therefore, several patents exist describing the isomerisation and cyclisation steps involved. In the first step, both olefinic double bonds of the primary Diels–Alder product **32** can isomerise, thereby creating several precursors **49**–**52** that, accept for **52**, are suited to undergo a second cyclisation as depicted in Scheme 7. After the following cyclisation step, the double bond of the racemic products obtained isomerises between α, β and γ [25].

**Scheme 8:**
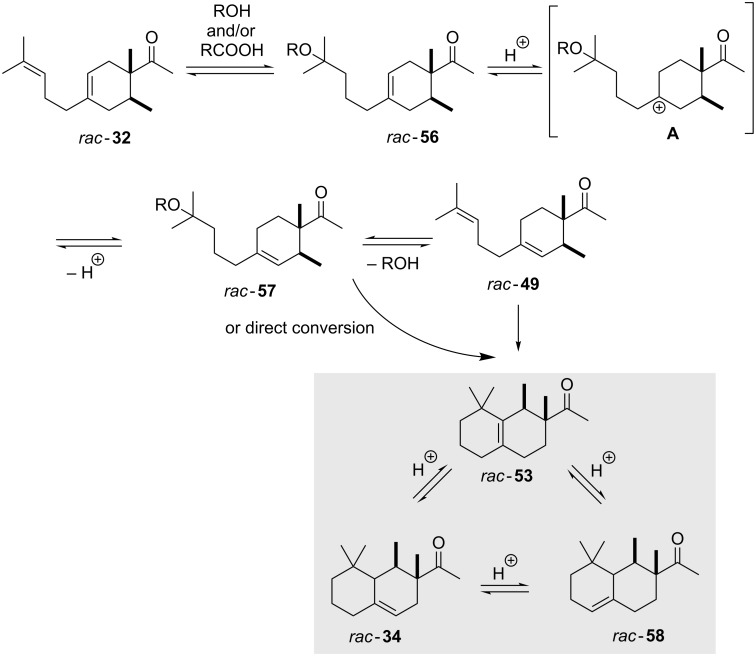
Isomerisation using additives such as alcohols or carboxylic acids. The product with the γ*-*positioned double bond is the desired Iso E Super Plus^®^ (**34**). Products **58** (α double bond) and product **53** (β double bond) are not desired [26].

Furthermore, Erman and co-workers from Millenium Speciality Chemicals Inc. described a process, which involves methanol and other alcohols or alternatively organic acids as nucleophilic additives that can reversibly be introduced and removed again (Scheme 8). Typically, methanol, ethanol, isopropanol and 2-methoxyethanol served as suitable alcohols. According to patent information di- or polyols can also serve as “dummy” additives. Alternatively, also acetic acid was suggested. Using this method, the desired Iso E Super Plus^®^ (**34**) concentration ranged from 5% to 7% as judged by GC analysis [26].

**Scheme 9:**
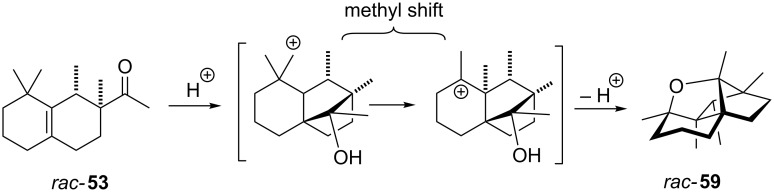
Iso E Super Plus^®^ (**34**) can undergo a third cyclisation to tetrahydrofuran **59** through compound *rac*-**53** [22].

Fráter and Schröder discovered that Iso E Super Plus^®^ (**34**) can undergo an additional cyclisation through compound *rac*-**53** (Scheme 9). This is initiated by the acid employed in the second step of the synthesis. Thus, the ketone is protonated and the highly electrophilic carbon atom reacts with the alkene moiety. The resulting tertiary carbocation undergoes a 1,2-methyl shift to yield a new cation, which in turn is nucleophilically trapped by the carbinol moiety. The resulting tetrahydrofuran **59** is chemically stable and this observation was used as rationale for the erosion of the isomeric ratio observed during prolonged reaction times.

In the same piece of work Fráter et al. investigated the influence of Brønstedt and Lewis acids on the formation of Georgywood^®^ (**35**). It was found that Lewis acids such as AlCl_3_ shift the equilibrium towards Georgywood^®^ (**35**) type products especially when employed in over-stoichiometric amounts. Using different Brønstedt acids, the ratios between the products obtained can change drastically [22].

## Conclusion and Outlook

**Figure 5:**
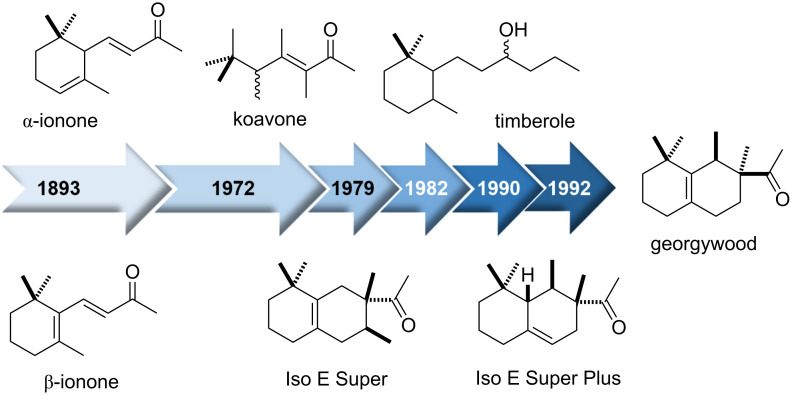
(Adapted from ref. [8]) Ionone (**30**, 1893, odour threshold: 0.8 ng L^−1^), koavone (1982, odour threshold: 75 ng L^−1^), Iso E Super^®^ (**33**) (1972, odour threshold: 500 ng L^−1^), timberole (1982, odour threshold: 26 ng L^−1^) Iso E Super Plus^®^ (**34**) (1990, odour threshold: 5 pg L^−1^) and Georgywood^®^ (**35**, 1996, odour threshold: 15 pg L^−1^–30 pg L^−1^) [9,15,33,35–38].

**Figure 6:**
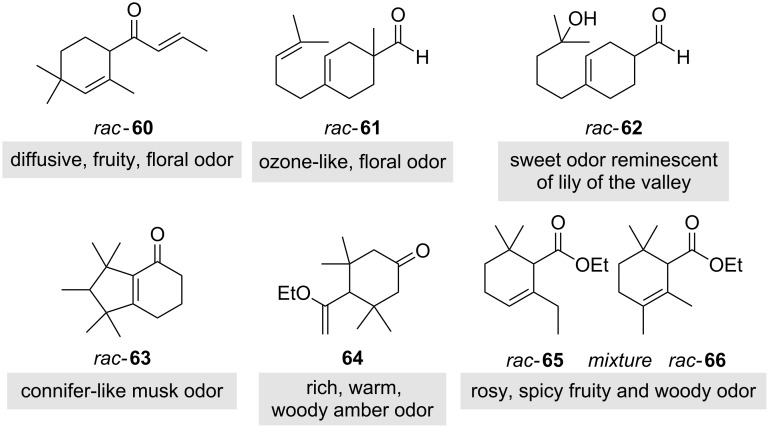
Branched, terpene-like cyclohexene derivatives, that are synthetic fragrance components: **60**: Iso damascone, **61**: Precyclemone B, **62**: Lyral^®^, **63**: Cashmeran^®^, **64**: Kephalis, and **65**, **66**: Givescone^®^.

Here, we presented a short story on Iso E Super^®^ and derivatives formed during synthesis, a group of molecules that has changed the perfume industry, but has its roots in the terpenoid ingredients of classical essential oils geranium and bergamot (Figure 5). Starting from ionone (**30**) an “evolutionary process” towards synthetic products with similar olfactory properties led to Iso E Super^®^ (**34**), Iso E Super Plus^®^ (**35**) and Georgywood^®^ (**35**), a development that took almost hundred years and saw koavone and timberole as intermediates. An analysis of today´s fine fragrances reveals that almost all of them combine synthetic scent molecules with traditional essential oils, despite the fact, that the ongoing consumer trend is towards natural ingredients. Avoiding synthetics like Iso E Super^®^ (**33**) would rule out many favourite scents. In fact, about 100 natural fragrance ingredients are known, but perfumers have more than 3,000 synthetic molecules at hand of which several examples **60**–**66** with terpene-like structures are listed in Figure 6. Noteworthy, the fragrance properties of synthetically-derived unnatural compounds commonly mimic those of natural products.

Biotechnology is another way to harness fragrance components be it enzymatic or microbial. Nowadays engineered microbes are at hand that, e.g., produce scents, such as patchouli, by fermenting sugar. Patchouli is a complex mixture of sesquiterpenes ((−)-patchoulol, (+)-norpatchoulenol, (+)-α-bulnesene, (−)-α-guajene, (−)-β-patchoulene and (−)-seychellene) with a slightly camphoraceous, woody balsamic odour [6].

**Scheme 10:**
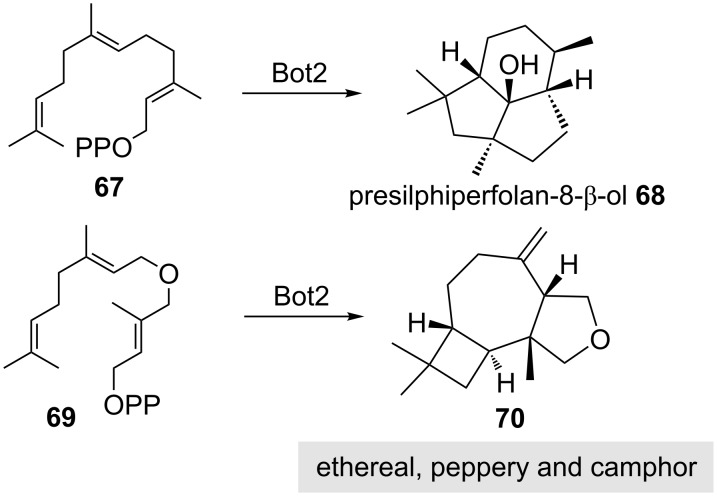
New unnatural terpenoid **70** from unnatural farnesyl pyrophosphate derivative **69** and comparison with natural biotransformation (**67→68**) and olfactory property of tetrahydrofuran **70** [39].

Enzymatic derivatisation of terpenes by means of biocatalysis is another opportunity to create new fragrance molecules or to achieve chiral resolution of racemates. The former process is commonly associated with oxidation reactions, while the latter process is often based on the action of lipases. Very recently, a new concept was disclosed that probed sesquiterpene cyclases to accept unnatural farnesyl pyrophosphates and generate unnatural cyclisation products with unusual backbones. Thus, in the presence of presilphiperfolan-8-β-ol synthase (Bot2) a novel tricyclic product **70** was obtained from unnatural farnesyldiphosphate ether **69**. The olfactory analysis revealed an ethereal, peppery and camphoric scent (Scheme 10) [39].

Future prospects of the fragrance industry will be linked with a bouquet of methods to broaden the platform of molecules with favourable olfactory properties. These include chemical synthesis, microbiology and molecular biology associated with biotechnology and combinations based on these methods. Hence also the most recent developments in synthetic biology will appear on the stage of the world of fragrances [40–41].
